# The Treatment of Infectious Diarrheas in Infancy

**Published:** 1916-08

**Authors:** W. W. Harper

**Affiliations:** Selma, Ala.


### THE TREATMENT OF INFECTIOUS DIARRHEAS IN INFANCY.

W. W. Harper, M. D., Selma, Ala.

To successfully treat the infectious diarrheas of infancy, one must separate the cases of intoxication from those of true dysentery. It must be determined also whether the infecting organism is the gas bacillus or the Flexner and its allies.

In the intoxication type, the baby becomes suddenly ill with vomiting and high fever. The stools are frequent, rather large, watery, slimy, green and very offensive. Blood and jelly-like mucous are not present. A chemical examination of the stool shows the liquid part to be very rich in albumin. This fact is important because it reveals a severe protein loss through osmosis into the intestinal canal. Again, this fact teaches the folly of prolonged initial starvation in the treatment. Formerly, these patients were starved under the belief that in this way the causative organisms were being starved. But it was found that when food was withheld for any considerable length of time, the germs attacked the rich albuminous intestinal esmosis (Morse) and continued the infection. The baby shows the intense toxemia by it's "dopy" appearance. The face is sallow, the eyes are hollow and the baby lies in a stupor —being restless when aroused. It is noted that the respirations are deep and sighing. Unless relieved at this stage, the respiration becomes more rapid and more labored (hyperphoca). The baby grows more restless, rolls the head from side to side and throws its arms about.

A chemical analysis of the urine will reveal acetone. Howland has shown that in most of those cases acidosis is the cause of death, and as practically every case of marked acidosis dies, the condition must be prevented by prompt and vigorous treatment of all cases of diarrhea. Give at once a dose of castor oil and if the stomach rejects it, give small, repeated doses of calomel until 1 to 3 grains are taken. In the first twenty-four hours flush the bowels every six hours with a 1 per cent. soda solution, —leaving in the bowel as much soda as possible. Prepare the soda solution as follows: Put a teaspoon full of soda bicarb in a pint of water which has been boiled. Do not boil soda bicarb as this converts it into the carbonate which is irritating. After allowing two hours for the oil to leave the stomach, give 10 to 30 grains (according to baby's age) of soda bicarb in 1 to 2 ounces of water every hour until urine becomes alkaline, and then every two hours until acetone disappears from urine. The soda can then be gradually reduced. Where the stomach refuses to retain the soda, give by vein 4 to 8 ounces (according to size of child) of a 2 per cent. soda and 5 per cent. glucose solution. This should be repeated every 8 hours if necessary. Mariot has shown that in these diarrheas there is rapid loss of sodium and that the blood is depleted of its carbonates. Therefore these patients are alkali tolerant and the alkali especially needed is the sodium bicarb.

During the period of initial starvation the patient should be made to take as much cool, sterile water as it was taking milk before becoming ill. If it refuses to take plain water, it will often take a lemonade or orangeade sweetened with saccharine (one tablet to a pint of water). Saccharine is ideal for this purpose as it has no therapeutic nor food value and does not ferment in the intestinal canal,—it simply sweetens. If the baby will not take water or cannot retain same, do not wait until the tissues become dessicated but relieve the toxemia and dessication by administering a hypodermoclysis of a 1 per cent. salt and 5 per cent glucose solution. Give 4 to 8 ounces every six to eight hours. This dilutes the toxins, stays acidosis and acts as an ideal diuretic. Instead of a hypodermoclysis, the Massachusetts General Hospital gives the solution intravenously,—using the longitudinal sinus. At the Hopkins, the solution is injected into the peritoneal cavity, but neither method is practical outside of a hospital, and without experience the technique is difficult. As soon as the initial purgative has freely acted, excessive peristalsis and watery movements should be controlled with frequent, small doses of paregoric, or, if necessary, with morphine by needle. The opiate is a two-edged sword and should be given with great caution and discontinued just as soon as possible. For the pallor, cold extremities and prostration, atropine—hypodermatically—(1-500 to 1,-1000 grain) acts well. It can be repeated as needed. In place of atropine, camphor (1 to 2 grains) in oil may be used. For a quickly acting stimulant, caffein is ideal, but its effects lasts less than 1 hour.

High temperature is best relieved by a five-minute tub bath, —beginning with a temperature of 100 which is quickly lowered to 95. This not only promptly reduces the temperature but the tepid water allays nervousness.

The writer has long since abandoned bismuth and all the so-called intestinal antiseptics and astringents, as he has never seen any good come from their use and he believes they are harmful by often upsetting an already irritated stomach. Do not practice long starvation, but you should remember that these cases are fat intolerant. Therefore, the fat per cent. in the nourishment should be kept low.

In breast fed infants, it is the writer's practice to resume nursing after twelve to eighteen hours' starvation. The directions are to nurse one minute out of each breast every four hours,—preceding each nursing with 2 drams of lime water and 1 to 2 ounces sterile water. This gives food which is low in fat and sugar, with a fair amount of proteid. Besides, the breast-fed receives from its mother certain immune bodies which help to overcome the disease. If, at the end of 24 hours, the child is progressing satisfactorily, the nursing period is increased one minute out of each breast. As long as the baby continues to improve, the time of nursing is increased every day one minute from each breast until the baby is nursing full time. After the primary starvation of 12 to 18 hours, the breast is indicated no matter what type of infection prevails. If the baby is bottle fed, the chances of recovery will be markedly increased if breast milk can be used even for a few days. If breast milk cannot be obtained, then the baby must be fed at the end of twenty-four hours' starvation upon carbohydrate of protein milk. While it is being starved, one should learn the type of infecting organism. If it is the Flexner or one of its allies, give every three hours 4 to 8 ounces of a 5 per cent. lactose solution. Twenty-four hours after that, for babies under six months the

5 per cent. lactose should be given in barley water. For babies between six and twelve months, add the lactose to thick gruel. For those over twelve months of age, these thick gruels may be alternated with crisp crackers, fresh dry toast or beaten biscuit. Give one ounce of bacillary milk four times a day to keep the intestinal canal sown with normal intestinal flora (lactic acid bacilli). As soon as the patient is temperature free and the bowels have thickened quite a bit, cautiously add boiled skim milk to the diet. When the patient is taking the normal amount of skim milk, begin replacing every few days one ounce of skim milk with one ounce of whole milk, but do not exceed a 3 per cent. fat, and watch the stools carefully for free fats.

If the infection is due to the gas bacillus, give 4 to 8 ounces of bacillary or protein milk every three to four hours. When the stools have become somewhat salve like, cereals should be cautiously added to the diet,—their use being discontinued if the temperature goes up, or if the stools become frequent and spongy.

Intoxication diarrheas usually run a short course and when the patient commences to improve, the recovery is prompt.

In the dysentery type the stools contain blood and jelly-like mucus. The bowel movement is preceded by tormina and is followed by tenesmus. While the baby looks toxic, the face is flushed and the eyes bright and not sunken. The respiratory balance is usually not disturbed and acetonuria is not so frequently found as in the intoxication type.

As a rule the stomach is not upset in the dysentery type and the baby generally takes enough water to prevent tissue dessication. Therefore, there is not that urgency for hypodermoclysis and intravenous injections of soda and other solutions as in intoxication diarrheas. However, soda bicarb should be given as a routine measure to prevent acidosis. Otherwise the treatment, both medical and dietetic is the same as that given for the intoxication form.

One must remember that in dysentery the intestinal mucosa is ulcerated and that recovery is slow and that relapses are frequent.

